# On-orbit electrical power system dataset of 1U CubeSat constellation

**DOI:** 10.1016/j.dib.2022.108697

**Published:** 2022-10-26

**Authors:** Adolfo Jara, Pooja Lepcha, Sankyun Kim, Hirokazu Masui, Takashi Yamauchi, George Maeda, Mengu Cho

**Affiliations:** Laboratory of Lean Satellite Enterprises and In-Orbit Experiments (LaSEINE), Kyushu Institute of Technology, 1-1, Sensui-cho, Tobata Ward, Kitakyushu City, Japan

**Keywords:** 1U CubeSat, Solar panel, Electrical power system, On-orbit dataset, BIRDS program

## Abstract

This article presents a database containing on-orbit data samples of the Electrical Power System (EPS) from 4 different 1U CubeSats belonging to the BIRDS constellation. The EPS is responsible for providing uninterrupted power to overall satellite both during sunlight and eclipse. The satellites are based on the BIRDS open-source standardized bus designed by Kyutech for research and education. BIRDS bus was used for six satellites that were delivered to ISS on board the Cygnus re-supply spacecraft launched by Antares rocket and released from International Space Station (ISS) into ISS orbit (altitude 400 km, inclination: 51.6°, duration: 92.6 min). The dataset contains the data of voltage (mV), current (mA) and temperature (Celsius) of the battery and solar panels attached to 5 sides of the satellite. This data is collected by the on-board computer every 90 seconds in nominal operation or every 10 seconds in fast sampling mode. The data is downloaded from the satellite memory by the ground station operators. Next, space engineering experts from Kyushu Institute of Technology have analysed the dataset to classify each data sample into normal or anomaly classes. This paper provides one datafile per satellite, that includes data from solar panels and battery since their deployment into orbit until the end of its life for the UGUISU, RAAVANA, and NEPALISAT satellites, first two showing a failure in one of their panels during more than two years of operation on-orbit. The TSURU satellite dataset includes data since its deployment into orbit and will continue to be collected until the end of its life. The dataset generated will be useful for 1U CubeSat, such as BIRDS platform, users, and satellite developers by using it as a reference to compare the behaviour of their Electric Power System under different operating scenarios and align their missions according to the available power on-orbit. At the same time, the dataset can help computer science researchers to build and validate new models for fault diagnosis and outlier detection.

**Specifications Table**

**Table:** 

Subject	Aerospace Engineering
Specific subject area	1U CubeSat Electric Power System
Type of data	Table
How the data were acquired	The data was acquired from the Electric Power System (EPS) of four different satellites orbiting in Low Earth Orbit with the inclination of 51.6 degree and the height from 400km or less. Each panel of the satellites has a LMP8640 current sensor to measure current data. The voltages from panels are measured by the AD7490 Analog to Digital Converter (ADC), while the temperature data is measure by a LMT84 temperature sensor. The battery current is measured by the ACS722LLCTR-05AB current sensor, the battery voltage is measured by the ADC of a PIC16F1789 microcontroller, and the battery temperature is measured by a G10K3976 thermistor.
Data format	Raw Cleaned Calibrated
Description of data collection	The satellites operators periodically send a command to execute the High Sampling Sensor Data Collection (HSSC) mission, the satellites collect the EPS data during one orbit (∼92.6 min). The sampling interval of UGUISU, RAAVANA, and NEPALISAT is 5 seconds, while for TSURU is 10 seconds. During the mission execution the data is saved in a MT25QL01Gb flash memory. Subsequently, the team downloaded the raw data to the ground station where it is converted to the actual values using the corresponding formulas for each magnitude from the datasheet of the sensors and manual sensor calibration.
Data source location	• Institution: Laboratory of Lean Satellite Enterprises and In-Orbit Experiments (LaSEINE), Kyushu Institute of Technology • City/Town/Region: Kitakyushu • Country: Japan
Data accessibility	Repository name: Mendeley Data Data identification number: DOI: 10.17632/8kp25ycf63.1 Direct URL to data: https://data.mendeley.com/datasets/8kp25ycf63/1
Related research article	A. Jara, B.H.B. Pangestu, A. Hanazawa, M. Cho, Performance Evaluation of Machine Learning Methods for Anomaly Detection in CubeSat Solar Panels, Appl. Sci. 12, (2022), 8634. https://doi.org/10.3390/app12178634.

## Value of the Data

• The dataset is useful and important because it includes on-orbit data collected by four different CubeSats that uses the same bus system design: NEPALISAT, RAAVANA, UGUISU and TSURU satellites. The data can be used to estimate energy available on-orbit for 1U CubeSat to assess the feasibility of the missions. The satellite design information has been put in public domain as BIRDS open-source standardized bus system to promote rapid and easy satellite development for educational and research purpose. The data can be used as a standard dataset to verify the on-orbit performance of Electrical Power System of the satellite developed using the BIRDS bus. NEPALISAT, RAAVANA and UGUISU were operated on orbit for more than two years until their re-entry to the atmosphere. TSURU is still operating on orbit and samples will continue to be collected until the end of life of the satellite.
• BIRDS platform users and satellites developers can benefit from the dataset by using it as a reference to compare their satellite Electric Power System operation. The dataset can assist in training space engineers to visualize and better understand the system behaviour under different operating scenarios. At the same time, the dataset can help computer science researchers to build and validate new models for fault diagnosis and outlier detection.
• The dataset can be used to train and/or validate supervised and unsupervised learning models.

## 1 Data Description

The presented data, which are also publicly available on Mendeley data repository (see details and link in the specification table above), include on-orbit data of solar panels and battery collected from the Electric Power System (EPS) of four different CubeSats as part of the Joint Global Multi-National Birds or BIRDS program, multi-national small satellite research and educational program led by the Kyushu Institute of Technology (Kyutech) in Japan [1]. The satellites use the BIRDS standardized bus, an open-source initiative by Kyutech designed for educational CubeSat projects [2].

The collection of EPS dataset was done using a PIC16F1789 microcontroller that reads sensor data from the external panels and battery of the satellite (discussed in next section). After the data is downloaded from the satellites at ground station they are saved in the corresponding directories, the directories are named as TSURU, RAAVANA, UGUISU, and NEPALISAT. Each directory is divided in spreadsheets named with the pattern ddmmyy.xls, where ddmmyy represents the date of the data collection. Each record file contains attributes defined with examples in Table 1.

**Table 1:** Explained example of the EPS dataset.

Column	Meaning	Example
Time stamp	The time in seconds in which the data sample was generated	159
Tpy_raw	Temperature value of the positive Y panel in HEX format (2 bytes)	067E
Tpy	Temperature value of the positive Y panel in calibrated format (°C)	3.74
Tpx_raw	Temperature value of the positive X panel in HEX format (2 bytes)	0635
Tpx	Temperature value of the positive X panel in calibrated format (°C)	11.85
Tmz_raw	Temperature value of the negative Z panel in HEX format (2 bytes)	06BE
Tmz	Temperature value of the negative Z panel in calibrated format (°C)	-3.36
Tmx_raw	Temperature value of the negative X panel in HEX format (2 bytes)	06C8
Tmx	Temperature value of the negative X panel in calibrated format (°C)	-4.47
Tpz_raw	Temperature value of the positive Z panel in HEX format (2 bytes)	05CD
Tpz	Temperature value of the positive Z panel in calibrated format (°C)	23.39
Vpy_raw	Voltage value of the positive Y panel in HEX format (2 bytes)	0CDF
Vpy	Voltage value of the positive Y panel in calibrated format (mV)	5029.00
Vpx_raw	Voltage value of the positive X panel in HEX format (2 bytes)	0D22
Vpx	Voltage value of the positive X panel in calibrated format (mV)	5131.26
Vmz_raw	Voltage value of the negative Z panel in HEX format (2 bytes)	0D06
Vmz	Voltage value of the negative Z panel in calibrated format (mV)	5128.21
Vmx_raw	Voltage value of the negative X panel in HEX format (2 bytes)	0CEA
Vmx	Voltage value of the negative X panel in calibrated format (mV)	5088.52
Vpz_raw	Voltage value of the positive Z panel in HEX format (2 bytes)	0D08
Vpz	Voltage value of the positive Z panel in calibrated format (mV)	5091.58
Ipy_raw	Current value of the positive Y panel in HEX format (1 bytes)	03
Ipy	Current value of the positive Y panel in calibrated format (mA)	13.98
Ipx_raw	Current value of the positive X panel in HEX format (1 byte)	2E
Ipx	Current value of the positive X panel in calibrated format (mA)	214.41
Imz_raw	Current value of the negative Z panel in HEX format (1 byte)	13
Imz	Current value of the negative Z panel in calibrated format (mA)	88.56
Imx_raw	Current value of the negative X panel in HEX format (1 byte)	06
Imx	Current value of the negative X panel in calibrated format (mA)	27.97
Ipz_raw	Current value of the positive Z panel in HEX format (1 byte)	18
Ipz	Current value of the positive Z panel in calibrated format (mA)	111.87
VBatt_raw	Voltage value of the battery in HEX format (1 byte)	A4
VBatt	Voltage value of the battery in calibrated format (V)	4.17
IBatt_raw	Current value of the battery in HEX format (2 bytes)	082E
IBatt	Current value of the battery in calibrated format (mA)	-131.99
TBatt_raw	Temperature value of the battery in HEX format (1 byte)	BB
TBatt	Temperature value of the battery in calibrated format (°C)	3.37

## 2 Experimental Design, Materials and Methods

**Fig. 1:**
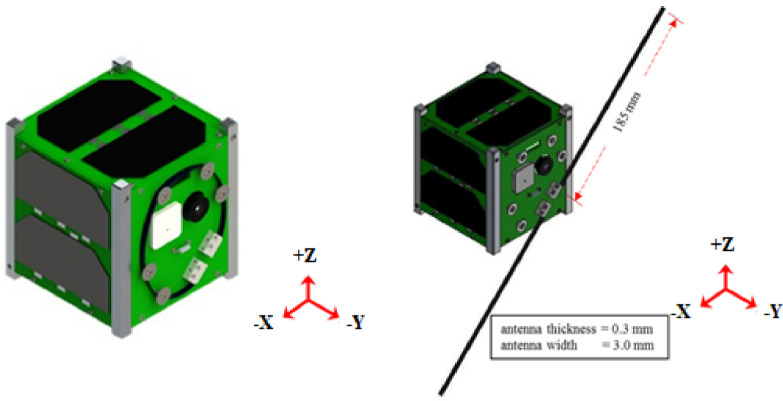
BIRDS platform external structure.

**Fig. 2:**
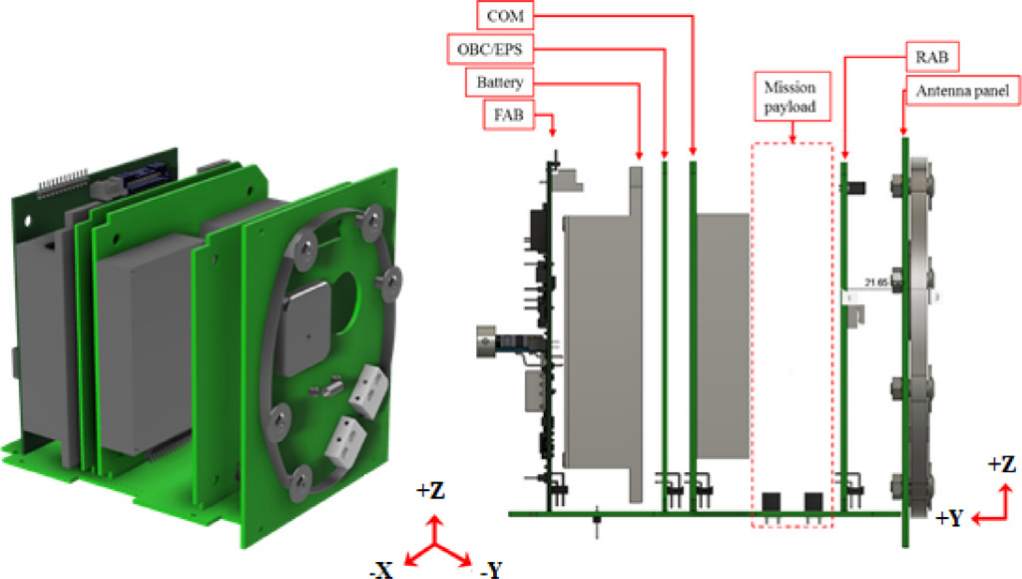
BIRDS platform internal structure.

The BIRDS satellite's platform conforms to 1U CubeSat standard [3] and are designed seeking low-cost and fast delivery [4]. The external dimensions are approximately (114 × 107 × 104) mm with an average mass of 1.3 kg. The satellite external view is shown in Fig. 1 below:

The bus system consists of the Front Access Board (FAB), the On-Board Computer (OBC)/EPS board, the Communication board (COM), and the Rear Access Board (RAB). The EPS elements are distributed in FAB Board and OBC/EPS board. All printed circuit boards (PCBs) are connected to a backplane board via 50-pin connectors. The satellite internal structure is shown in Fig. 2 below:

The RAB is used for programming and monitoring each mission microcontrollers. Available 2 mission boards are utilized for the missions of the satellites.

The FAB board is used for programming and monitoring the Main PIC, FAB PIC, RESET PIC and COM PIC microcontrollers. The FAB board uses a PIC16F1789 that fulfils the purpose of collecting housekeeping data from solar panels and battery. The FAB PIC receives commands and sends housekeeping data to the Main PIC using the UART communication protocol.

The OBC includes the main PIC and a Reset PIC enabled as a watchdog. The main PIC is a PIC18F67J94, which works as the brain of the CubeSat and its tasks are to collect and store the housekeeping data, send the command for the antenna deployment, process the uplink commands and execute the missions accordingly. The main PIC has a 1Gb MT25QL01Gb flash memory divided into 2048 sectors of 64 KB that are assigned according to the needs of each mission. A PIC16F1789 is used as a RESET PIC. The RESET PIC keeps counting the time elapsed since the deployment into orbit, resets the Main PIC in case of a single event latch up and monitors the current drawn over the power lines. The main PIC and the reset PIC communicate through UART.

**Fig. 3:**
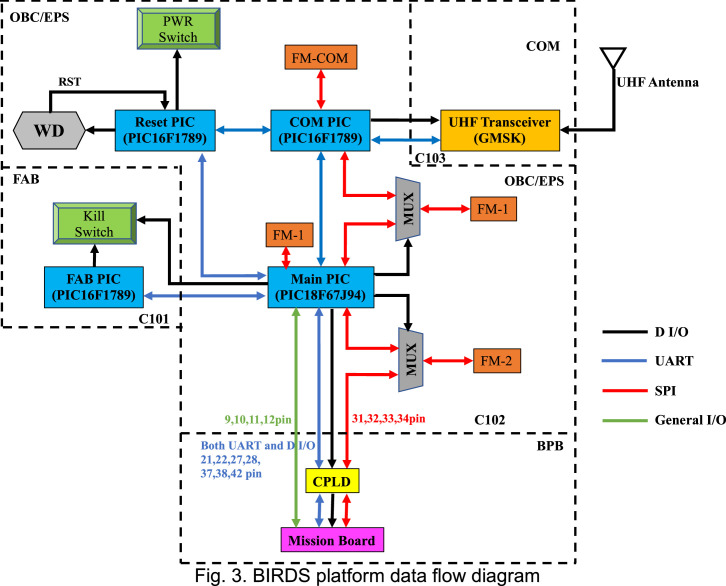
BIRDS platform data flow diagram.

COM has a PIC16F1789 microcontroller located on the OBC board. COM PIC functions are receiving command from the ground station and sending those commands to the main PIC, creating the CW format, and transmitting those data periodically to the ground. COM PIC communicates with Main PIC through UART protocol. COM PIC has its own MTQ25QL01Gb 1Gb flash memory. A dipole antenna is mounted on the –Y panel. Telemetry data and CW data are sent to the ground station by communicating through this antenna. The frequencies used for uplink and downlink are in the amateur radio band. The downlink frequency is 437.375 MHz (Fig. 3).

### 2.1 Electrical power system description

**Fig. 4:**
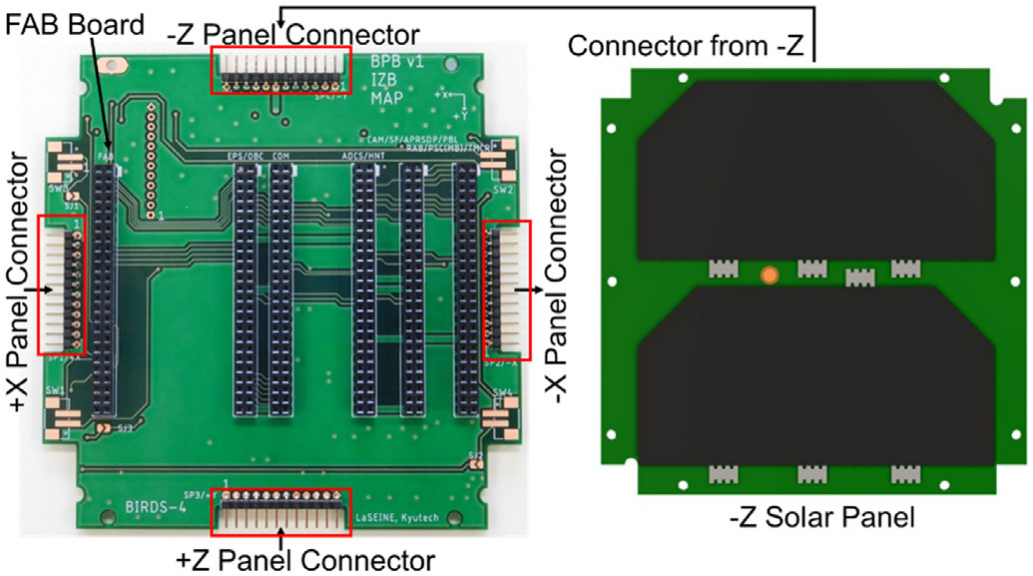
Solar panel routing to FAB through backplane.

In BIRDS bus, the solar cells responsible for power generation are attached on the five sides of the satellite (+X, +Y, +Z, -X, -Z), with two cells connected in series and the string of two cells connected in parallel from all sides of the satellite. The specific solar cell (AZURESPACE 3G30A) has an ideal maximum power generation of 1.2 W with an open circuit voltage (OCV) of 2.690 V. The solar panel output is routed to FAB board via backplane using through hole connectors as shown in Fig. 4. The solar cells are attached to the solar panels in-house using room-temperature-vulcanizing (RTV) silicone glue, RTV S-691, and conductive glue called DOTITE.

The BIRDS bus uses Linear Technology Corporation's LTC3119 as the battery charger, given its ability to track the maximum power point of solar cells. The voltage generated from the solar panels is fed to the battery charging IC through blocking diodes, voltage and current sensors and the separation switches which are a requirement for safety review to prevent inadvertent charging of the satellite. The output voltage of the DCDC converter is designed to be 4.2 V nominal which is the maximum charging voltage of the battery pack. Therefore, even if an excessive voltage is generated from the solar panel side with respect to the battery, the voltage is stepped down by the DCDC converter to 4.2 V and prevents overcharging.

**Fig. 5:**
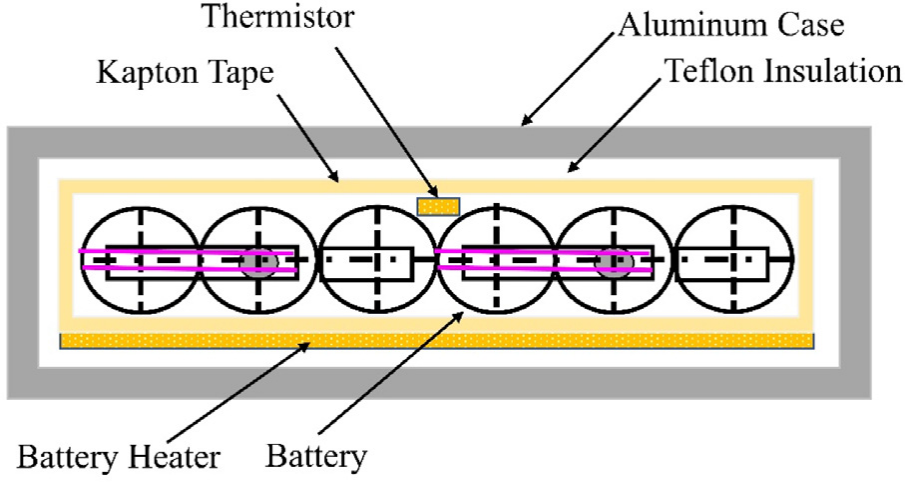
Battery box cross-sectional view.

The energy generated is stored in a battery pack of six rechargeable Eneloop Nickel Metal Hydride (NiMH) batteries with a minimum capacity of 1900 mAh per battery arranged in a 3-series and 2-parallel configuration. The specifications of a single battery cell is detailed in Table 2. The battery pack is assembled into a battery box as shown in Fig. 5.

**Table 2:** Battery specification.

Single Cell	
Model number	BK-3MCCE
Rated capacity	2000 mAh
Nominal voltage	1.2 V
Weight	26 g
Size	ø: 14.5 x L: 50.5 mm

A thermistor is placed between batteries that measures the temperature of the battery. The thermistor is connected to the FAB board using a harness and temperature is read by the FAB PIC. Since the batteries capacity is strongly affected by cold temperatures, the battery pack is wrapped with Kapton tape, and a polyimide heater that helps to maintain the thermal balance. The battery heater is turned off for this experiment. A layer of Teflon is placed inside of battery box before placing the battery pack.

The batteries were selected through a series of charge-discharge cycles before and after space environment tests such as vacuum leak tests and vibration test. For making the battery packs, only the batteries that show minimal deviation in characteristics were selected. The batteries are grouped based on their internal impedance to avoid failures due to cell mismatch.

**Fig. 6:**
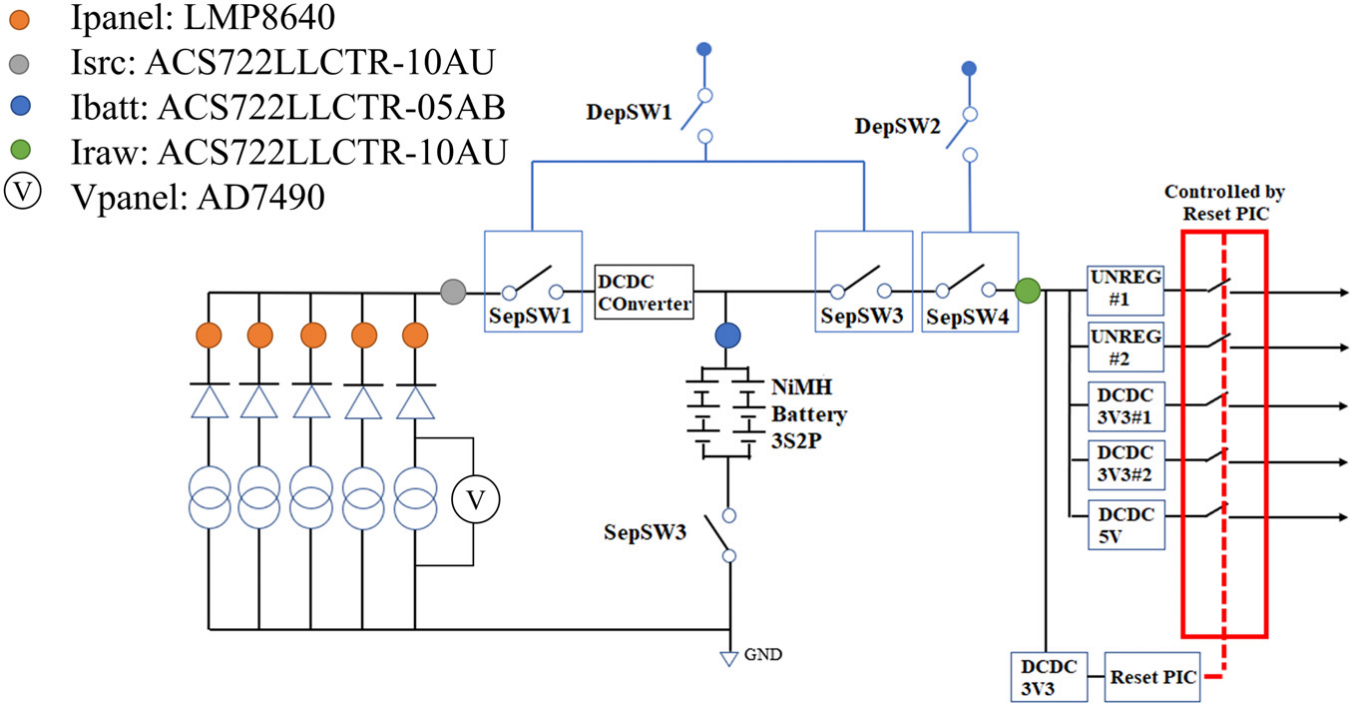
BIRDS-4 satellite EPS block diagram.

TPS63020-Q1 voltage regulators are used to convert bus voltage into respective regulated voltage lines and provide power to all missions and satellite subsystems. The power lines of the CubeSat are controlled by the Reset PIC. Two 3.3 V lines, one 5 V line and two unregulated lines are used. The unregulated line #2 is used for the deployment of the antennas and unregulated line # 1 as the power supply for the COM transceiver. The block diagram of BIRDS EPS is shown in Fig. 6 below:

The block diagram also shows the location of current and voltage sensors in the circuit. The current sensor for the individual solar panels (+X, +Y, +Z, -X, -Z), LMP8640 is placed in series to the output of solar panels, it is indicaled as Ipanel in Fig. 6. The voltage sensor is placed before the blocking diode to accurately measure the solar panel voltage without the influence of the bus voltage, indicated as Ⓥ in Fig. 6. The battery current sensor (ACS722LLCTR-05AB) is a bidirectional current sensor that can measure the charging and discharging current of the battery. The charging current is set as negative value and the discharging current is set to be a positive value. The total voltage of battery pack is measured using the ADC of the PIC microcontroller.

### 2.2 Experiment scenario

**Fig. 7:**
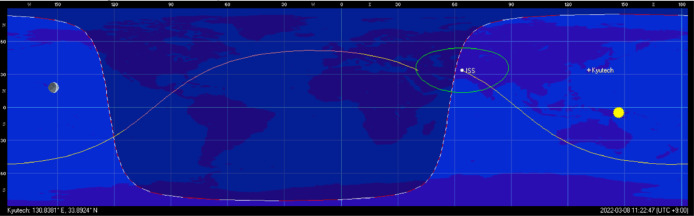
International space station orbit.

BIRDS satellites were delivered to ISS on board the Cygnus re-supply spacecraft launched by the Antares rocket. The BIRDS constellation was released from International Space Station (ISS) into ISS orbit as shown in Fig. 7 (altitude 400 km, inclination: 51.6 °, duration of one orbit: 92.6 min). This results in a beta angle that varies between ±75.1 ° over the course of a year, affecting the percentage of time that the satellites experience sunlight and eclipse, and hence determines the solar panels power generation and temperature profile. Since no attitude control is applied, the satellites are in free rotation at approximately 3 deg/s on each axis. The data were collected during the nominal operation of the satellite.

### 2.3 Data collection

**Fig. 8:**
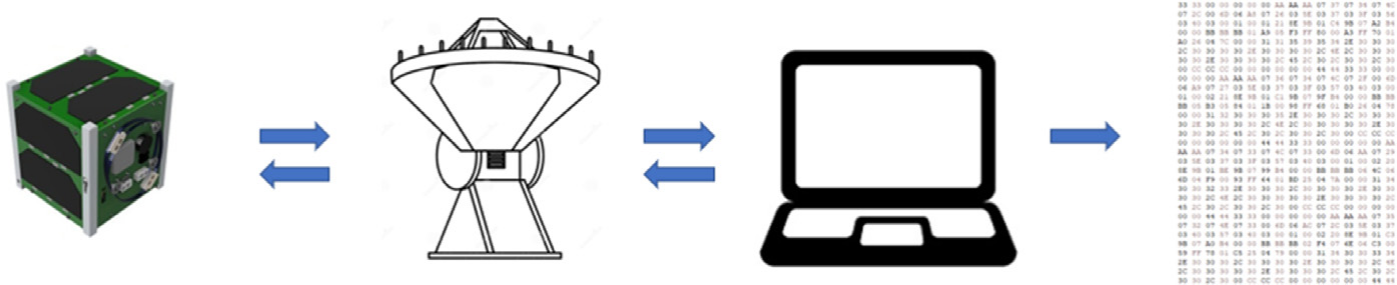
Command uplink and data downlink process.

The BIRDS team operators periodically execute the High Sampling Sensors data Collection mission (HSSC) to collect the satellite housekeeping data during one orbit. The command to execute the mission is sent from the ground station located at Kyushu Institute of Technology, Japan. Fig. 8 shows the command uplink and data downlink process. After the satellite receives the command, the FAB microcontroller starts the data collection process. The sampling interval is 10 s for the TSURU satellite and 5 s for NEPALISAT, RAAVANA and UGUISU satellites. Subsequently, the team downloads the data from the satellite when it passes over the ground station, where it is converted to the actual values using the corresponding formulas for each magnitude in the datasheet of the sensors and manual sensor calibration.

## Ethics Statements

We declare that the manuscript adheres to Ethics in publishing standards and the submitted dataset is the real data recorded in the experiment.

## CRediT Author Statement

**Adolfo Jara:** Methodology, Software, Data curation, Writing – original draft preparation; **Pooja Lepcha**: Software, Writing – review & editing; **Sangkyun Kim**: Conceptualization, Design, and Investigation; **Hirokazu Masui:** Testing and Documentation; **Takashi Yamauchi**: Safety Review Documentation; **George Maeda:** Reviewing and Editing; **Mengu Cho:** Conceptualization, Supervision, Reviewing and Editing.

## Declaration of Competing Interest

The authors declare that they have no known competing financial interests or personal relationships that could have appeared to influence the work reported in this paper.

## Data Availability

On-orbit Electrical Power System Dataset of 1U CubeSat constellation for Machine Learning Models (Original data) (Mendeley).
